# Organic Diets: Lu et al. Respond

**Published:** 2006-04

**Authors:** Chensheng Lu, Kathryn Toepel, Rene Irish, Richard A. Fenske, Dana B. Barr, Roberto Bravo

**Affiliations:** Rollins School of Public Health, Emory University, Atlanta, Georgia, E-mail: clu2@sph.emory.edu; Department of Environmental and Occupational Health Sciences, University of Washington, Seattle, Washington; National Center for Environmental Health Centers for Disease Control and Prevention, Atlanta, Georgia

Avery is concerned that the language used in our recent article (Lu et al. 2005), as well as in an earlier article (Curl et al. 2003), may be used to mislead the public regarding the relative safety of organic foods compared with foods derived from crops treated with pesticides.

We agree that communication of scientific information in general, and health risk information in particular, requires a careful choice of words. However, Avery’s analysis misconstrues the current scientific debate regarding children’s exposure to pesticides and misrepresents our work, thereby contributing to the public misunderstanding of this important issue.

In fact, we did choose our words carefully, and they reflect the essential findings of our studies. In regard to our earlier publication (Curl et al. 2003), we provided a detailed dose estimation to support our conclusion that consumption of organic fruits, vegetables, and juices in the study population would shift exposure from a range of uncertain risk to a range of negligible risk. In regard to the more recent study (Lu et al. 2005), our data clearly support the conclusion that organophosphorus (OP) pesticide exposures are dramatically reduced when organic foods are substituted for conventional foods. Our statement that children who consume organic foods would likely have a lower probability of neurologic health risks is consistent with current understandings of dose–response relationships. In other words, how could we argue that children with OP pesticide exposures have the same neurologic health risks as children whose urine contain no OP pesticide metabolites?

The assessment of health risks associated with neurotoxic chemicals such as OP pesticides is a complex analysis that includes substantial uncertainty. A child may be exposed to dozens of OP pesticides simultaneously through the diet, as well as through use of these pesticides around the home or in schools. The Food Quality Protection Act (1996) requires the U.S. Environmental Protection Agency to evaluate both children’s aggregate exposure (multiple exposure pathways for a single pesticide) and cumulative risk (potential health effects from exposure to multiple compounds that have a common mechanism of toxicity). Thus, current scientific investigations have focused on the relative contributions of specific exposure pathways and have attempted to examine exposure to multiple compounds. Our recent articles provide new information regarding the dietary exposure pathway for several OP pesticides.

Avery’s criticism of our work by focusing on a single OP pesticide ignores the central thrust of the Food Quality Protection Act (1996), as well as the scientific advances that have taken place over the past 10 years. We share Avery’s concern with the judicious use of language in regard to public communication of pesticide health risks; all of us—including Avery—should follow this advice.
